# Supplementary material for: ADELLE: A global testing method for trans-eQTL mapping
Source: PLoS Genet. 2025 Jan 10;21(1):e1011563. doi: 10.1371/journal.pgen.1011563 (PMC11756770; doi:10.1371/journal.pgen.1011563)
Supplement: S1 Text — Detailed description of the methods, including a model for Z, regularization of the sample covariance matrix, the beta-binomial approximation, pre-computation for the ADELLE test, assessment of type 1 error with Monte Carlo p-values, and generation of the correlation matrix for simulations. Additional results consist of numeric power results corresponding to Figs 2 and 3, power results for ADELLE with different choices of q, and significant trans eQTL detections by ADELLE in a region of Chrom 12 in the mouse AIL dataset. (PDF) [file pgen.1011563.s001.pdf]

S1 Text

Model for Z

In an eQTL mapping study in which $\tilde{D}$ expression traits and M genome-wide SNPs are observed on each of N individuals, define G to be the $M \times N$ genotype matrix for the SNPs and Y to be the $\tilde{D} \times N$ phenotype matrix for the expression traits. Let Z denote the $\tilde{D} \times M$ matrix of test statistics, where Z_{dm}, the (d, m)th entry of Z, is the test statistic for association between trait d and SNP m, and where each Z_{dm} is assumed to be standard normal under the null hypothesis of no association between trait d and SNP m. In data sets collected to date, it is common that $N \lll \min(\tilde{D}, M)$, i.e., there are many more expression traits and SNPs than there are individuals in the study.

In the simplest case, suppose that there are neither confounding covariates nor important population structure in the trait models. Then under the null hypothesis that none of the SNPs are eQTLs for any of the traits, the N columns of Y are assumed to be i.i.d. draws from $N_{\tilde{D}}(\mu_{trait}, V_{trait})$, where μ_{trait} is the $\tilde{D} \times 1$ vector of trait means and V_{trait} is the $\tilde{D} \times \tilde{D}$ trait covariance matrix, and the N columns of G are assumed to be independent, each having mean vector μ_{geno} of length M and $M \times M$ covariance matrix V_{geno}. Furthermore, suppose that Z_{dm} is obtained as the t-statistic for testing significance of SNP m in a simple linear regression of trait d on SNP m, for $1 \leq d \leq \tilde{D}, 1 \leq m \leq M$. Then for reasonably large N (even when $N \lll \min(\tilde{D}, M)$), the covariance structure of the elements of Z is given by

$$\mathrm{Cov}(\mathrm{vec}(Z)) = C_{geno} \otimes C_{trait}, \tag{1}$$

where C_{geno} is the correlation matrix derived from V_{geno} and C_{trait} is the correlation matrix derived from V_{trait}. A consequence of this model is that each row of Z has covariance matrix C_{geno} and each column of Z has covariance matrix C_{trait}. In the more general setting with a linear mixed model for the trait, including covariates, the model in Eq 1 becomes approximate rather than exact.

Since each element of Z is marginally standard normal and they have the correlation structure in Eq 1, it would be tempting to suppose that the joint distribution of the elements of Z must be multivariate normal, and to suppose that therefore Z must have the matrix normal distribution $\mathrm{MN}_{\tilde{D},M}(\mathbf{0}, C_{trait}, C_{geno})$. However, this is false. In fact the distribution of Z is a mixture of matrix normal distributions, where each component of the mixture has mean 0 and variance 1 for every element of Z, but where different mixture

components have different correlation matrices for $\mathrm{vec}(Z)$. One way to specify the distribution of Z in this case is to note that conditional on G, Z does have a matrix normal distribution in large samples:

$$Z|G \sim \mathrm{MN}_{\tilde{D},M}(\mathbf{0}, C_{trait}, \hat{C}_{geno}) \tag{2}$$

where $\hat{C}_{geno}$ is the $M \times M$ sample correlation matrix of the given G, which is a matrix of rank $N-1$. From this formulation, it can be seen that each Z is only of rank $N-1$, which makes sense, because there are only N observations of each phenotype and genotype on which the entire matrix Z is based. It can also be seen that the unconditional distribution of Z will be a mixture of matrix normals, where we mix over G. We note that it is also true in this case that

$$Z|Y \sim \mathrm{MN}_{\tilde{D},M}(\mathbf{0}, \hat{C}_{trait}, C_{geno}), \tag{3}$$

where $\hat{C}_{trait}$ is the $\tilde{D} \times \tilde{D}$ sample correlation matrix of the given Y, so $\hat{C}_{trait}$ is of rank $N-1$.

In this simple setting, $\Omega = C_{trait}$ where Ω is as defined in the main text (e.g. main text Eq 6), and we could directly estimate C_{trait} by first forming the sample correlation matrix $\hat{C}_{trait}$ for the $\tilde{D}$ traits. However, because typically $N \ll \tilde{D}$, the estimate $\hat{C}_{trait}$ is low rank. We, therefore, regularize it by using the shrinkage estimator [1] $\widehat{\Omega} = w\hat{C}_{trait} + (1-w)I$ as described below in subsection **Regularization of sample covariance matrix**.

Other approaches are possible. For example, one could consider a more general null model for Z:

$$Z|G \sim \mathrm{MN}_{\tilde{D},M}(\mathbf{0}, \Omega, C_G), \tag{4}$$

where C_G is a $M \times M$ correlation matrix that is a function of G and has rank $N-1$ (or, more generally, $\mathrm{rank}(C_G) = N-k-1$ if k PCs, PEER factors, or other covariates have been regressed out of the expression traits), and Ω is a $\tilde{D} \times \tilde{D}$ correlation matrix, where $\mathrm{rank}(\Omega) = \mathrm{rank}(C_{trait})$, which we assume is equal to $\tilde{D}$. Properties of this model are that Z is of rank $N-k-1$, every column of Z has covariance matrix Ω and every row of Z has covariance matrix C_G.

A simple (but low-rank) estimator for Ω in this model would be the sample correlation matrix for the rows of Z, given by $\mathrm{cov2cor}(Z(I_M - M^{-1}\mathbf{1}_M\mathbf{1}_M^T)Z^T$, where cov2cor is the function that maps a symmetric positive semi-definite matrix A with positive diagonal elements to a matrix of the same size with (i,j)th element $A_{ij}/\sqrt{A_{ii}A_{jj}}$. If C_G were available, a more efficient (but still low-rank) estimator could be obtained by both decorrelating and centering the rows of Z to obtain $\mathrm{cov2cor}(Z(C_G^- - C_G^-\mathbf{1}_M(\mathbf{1}_M^T C_G^- \mathbf{1}_M)^{-1}\mathbf{1}_M^T C_G^-)Z^T)$, where C_G^- is the Moore-Penrose generalized inverse of the (singular) matrix C_G. More generally, suppose we used

matrix $\tilde{C}^-$ in the estimator $\hat{\Omega}_1 = \text{cov2cor}(Z\tilde{C}^- Z^T)$, where $\tilde{C}^-$ is a symmetric, positive semi-definite matrix.

To decide which choice of $\tilde{C}^-$ to use in the estimator $\hat{\Omega}_1$, we could consider the following simple setting for estimation of a single correlation value ρ. Suppose $(X_1, Y_1), \ldots, (X_n, Y_n)$ are i.i.d. bivariate normal with mean vector $(0, 0)$ and with 2×2 covariance matrix having both diagonal elements equal to 1 and both off-diagonal elements equal to ρ. Then, if we let $\rho_{d1,d2}$ denote the $(d1, d2)$th entry of Ω, where $d1 \neq d2$, in large samples the $(d1, d2)$th entry of $\hat{\Omega}_1$ would be approximately unbiased for $\rho_{d1,d2}$ with variance $(1 - \rho^2_{d1,d2})^2 \text{tr}(C_G\tilde{C}^- C_G\tilde{C}^-)/[\text{tr}(C_G\tilde{C}^-)]^2$, which is minimized when $\tilde{C}^- = C_G^-$, in which case it becomes $(1 - \rho^2_{d1,d2})^2/\text{rank}(C_G)$. However, to increase the robustness of the estimator, we would usually prefer to choose a $\tilde{C}^-$ that is orthogonal to 1_M, so that we are also centering Z. Within this class of estimators, the variance is minimized when $\tilde{C}^- = C_G^- - C_G^-\mathbf{1}_M(\mathbf{1}_M^T C_G^- \mathbf{1}_M)^{-1}\mathbf{1}_M^T C_G^-$, which leads to the second low-rank estimator suggested above. The resulting variance of the estimator is $(1 - \rho^2_{d1,d2})^2/(\text{rank}(C_G) - 1)$.

Practical choices one could consider would be $\tilde{C}^- = I - M^{-1}1_M 1_M^T$ (which makes $\hat{\Omega}_1$ the sample correlation matrix) or $\tilde{C}^- = \hat{C}^-_{geno} - \hat{C}^-_{geno}\mathbf{1}_M(\mathbf{1}_M^T\hat{C}^-_{geno}\mathbf{1}_M)^{-1}\mathbf{1}_M^T\hat{C}^-_{geno}$, where $\hat{C}_{geno}$ is the sample correlation matrix of G, which would be the optimal choice among all $\tilde{C}^-$ that are orthogonal to 1_M, in the special case of simple linear regression. Given $\hat{\Omega}_1$, which is generally of rank $N - k - 1$, we could apply regularization to obtain full-rank $\widehat{\Omega}$ as in the next subsection. However, a major drawback of this alternative approach to estimating $\widehat{\Omega}$ is that the entire matrix Z is required, while in practice, summary statistics are often only available for a subset of the Z matrix. Because of this drawback, we do not give further consideration to the alternative approach. In contrast, the Ω estimation approach described in the main text is more widely applicable, because it can be used when only a subset of the Z matrix is available, as long as the available summary statistics include the sample covariance of the trait values.

Regularization of sample covariance matrix

To estimate Ω, we first directly estimate C_{trait} by forming the sample correlation matrix $\hat{C}_{trait}$ for the $\tilde{D}$ traits. However, because typically $N \ll \tilde{D}$, the estimate $\hat{C}_{trait}$ will be low rank. We, therefore, regularize it by using the shrinkage estimator [1] $\widehat{\Omega} = w\hat{C}_{trait} + (1 - w)I$. We now describe how we choose the regularization parameter w.

For the beta-binominal approximation defined in the next subsection, from Eq 10 below it can be seen that the leading term of the beta-binomial approximation to the quantity $V_0[S(c)]$ needed for l-value calculation depends on $\widehat{\Omega}$ through the quantity $O_2(\widehat{\Omega})$, where for any $D \times D$ positive semi-definite matrix M we define $O_2(M) = \sum_{1 \leq k < l \leq D}(M_{kl})^2$. Therefore, in our regularization procedure, we focus on choosing w that leads to accurate recovery of $O_2(\hat{C}_{trait})$ in simulated replicates.

We perform a search over $w \in (0, 1)$, and for each choice of w, we perform the following steps: (1) form

$\widehat{\Omega} = w\hat{C}_{trait} + (1-w)I_D$; (2) sample 4 replicates of the $D \times N$ matrix Y from $MN(0, \widehat{\Omega}, I_M)$ to obtain $Y^{(1)}, Y^{(2)}, Y^{(3)}$, and $Y^{(4)}$; (3) for $i = 1, 2, 3, 4$, let $\hat{C}^{(i)}$ denote the sample trait covariance matrix based on replicate $Y^{(i)}$; (4) compare $O_2(\hat{C}_{trait})$ to $.25 \sum_{i=1}^{4} O_2(\hat{C}^{(i)})$, and choose w to minimize the absolute difference between these two quantities.

Beta-binomial approximation

In this subsection, we describe the beta-binomial approximation [2] used to calculate values of $F_{(d)}$, the cdf of $\pi_{(d)}$, for $d = 1, \ldots, qD$, which is needed for obtaining the l-values used in forming the ADELLE test statistic. From Eq 8 in the main text, we have for the l-value

$$l_d(h) \equiv F_{(d)}(h) \equiv P_0(\pi_{(d)} \leq h) = P_0(S(-\Phi^{-1}(h/2)) \geq d), \tag{5}$$

where $S(c) = \sum_{d=1}^{D} \mathcal{I}\{|Z_d| \geq c\}$. If $\Omega = I$, then for $c \geq 0$, $S(c)$ has the null distribution of a Binomial$(D, 2\Phi(-c))$ random variable, and

$$F_{(d)}(h) = 1 - \sum_{j=0}^{d-1} \binom{D}{j} h^j (1-h)^{D-j}, \quad h \in [0,1], \tag{6}$$

which is the CDF of a Beta$(d, D+1-d)$ distribution. When $\Omega \neq I$, the null mean of $S(c)$ will remain the same, $E_0[S(c)] = 2D\Phi(-c)$, however the null variance is

$$\begin{aligned} V_0(S(c)) &= V_0\left(\sum_k \mathcal{I}\{|Z_k| \geq c\}\right) \\ &= 2D\Phi(c)(1-2\Phi(c)) + 2\sum_{i=1}^{D-1}\sum_{j=i+1}^{D} \text{cov}\left(\mathcal{I}\{|Z_i| \geq c\}, \ \mathcal{I}\{|Z_j| \geq c\}\right) \\ &> 2D\Phi(c)(1-2\Phi(c)) \end{aligned}$$

Where the last inequality follows from the positive correlation of the magnitudes of any correlated, mean-zero, bivariate Gaussians.

Following the method proposed by [2] (see also [3]), we approximate the distribution of $S(c)$ with a beta-binomial distribution $BB(D, a, b)$. The beta-binomial is the distribution formed from the binomial distribution when the success probability is drawn from a beta distribution. It is convenient to reparameterize the beta-binomial in terms of parameters $\lambda = \frac{a}{a+b}$ and $\gamma = \frac{1}{a+b}$. If X is drawn from a BB$(D, \ \lambda, \ \gamma)$, where

$\lambda > 0$, $\gamma > 0$, and D is a positive integer, then X has the following properties:

$$f_{D,\lambda,\gamma}(x) \equiv P(X = x) = \binom{D}{x} \frac{B(\frac{\lambda}{\gamma} + x, \frac{1-\lambda}{\gamma} + D - x)}{B(\frac{\lambda}{\gamma}, \frac{1-\lambda}{\gamma})}, \tag{7}$$

$$\lambda = E(X)/D, \text{ and} \tag{8}$$

$$\frac{\gamma}{1+\gamma} = \frac{V(X) - D\lambda(1-\lambda)}{D(D-1)\lambda(1-\lambda)}, \tag{9}$$

where $B(a, b)$ is the beta function.

To approximate the distribution of $S(c)$ we choose λ and γ by the method of moments. Barnett et al. [2] derived the following:

$$\begin{gathered} E_0\left[S\left(c\right)\right] = 2D\Phi(-c) \\ V_0\left[S\left(c\right)\right] = D\left[2\overline{\Phi}(c) - 4\overline{\Phi}^2(c)\right] + 4D(D-1)\phi^2(c)\textstyle\sum_{i=1}^{\infty}\mathcal{H}_{2i-1}^2(c)\rho(2i)/(2i)! \end{gathered} \tag{10}$$

Where $\mathcal{H}_i$ are the Hermite polynomials, $\rho(i) = \frac{2}{D(D-1)}\sum_{1\le k<l\le D}\left(\Omega_{kl}\right)^i$, where Ω_{kl} is the (k, l)th entry of Ω, and ϕ and $\overline{\Phi}$ are the density and survivor functions of a standard normal, respectively. Note that Eqs 8, 9, and 10 imply that once λ is set by the method of moments, knowing λ determines c which determines $V_0\left[S\left(c\right)\right]$ and thus determines γ. So we will drop the writing of γ (and D) below to simplify notation, and define $f_\lambda(x)$ to be the beta-binomial density with parameters (D, λ, γ), where λ and γ are related by the equations $\lambda = 2\Phi(-c)$ and

$$\frac{\gamma}{1+\gamma} = \frac{V_0[S(c)] - D\lambda(1-\lambda)}{D(D-1)\lambda(1-\lambda)},$$

with $V_0[S(c)]$ given in 10.

Applying this to Eq 5,

$$
\begin{aligned}
l_d(h) \equiv F_{(d)}(h) &= P_0\left[\pi_{(d)} \leq h\right] \\
&= P_0\left[S\left(-\Phi^{-1}\left(h/2\right)\right) \geq d\right] \\
&= 1 - \sum_{k=0}^{d-1} P_0\left[S\left(-\Phi^{-1}\left(h/2\right)\right) = k\right] \\
&\approx 1 - \sum_{k=0}^{d-1} f_h\left(k\right) \\
&= \sum_{k=d}^{D} \binom{D}{k} \frac{B(\frac{\lambda}{\gamma}+k, \frac{1-\lambda}{\gamma}+D-k)}{B(\frac{\lambda}{\gamma}, \frac{1-\lambda}{\gamma})} \\
&\equiv \hat{F}_{(d)}(h) \equiv \hat{l}_d(h). \qquad (11)
\end{aligned}
$$

Pre-computation for the ADELLE test

Our ADELLE method lends itself to a pre-computation to reduce computation time when it will be applied to a large number of SNPs and a large number of traits. Suppose we observe M SNPs along with the D traits. Then, from Eq 11, calculating M ADELLE statistics involves $M \times \sum_{j=1}^{qD}(D-j+1) \approx MqD^2$ evaluations of a beta-binomial probability mass function $f_h(k)$. However, we also see that computation of $\hat{l}_1(h)$ necessarily also computes all the l-values $\hat{l}_1(h), \ldots, \hat{l}_D(h)$.

This motivates a computationally efficient strategy for approximating the function $\hat{F}_{(d)}(h)$ for arbitrary h and $1 \leq d \leq qD$ by pre-computing a $qD \times H$ matrix of l-values, where H is the number of values of h in the grid. We choose a set of pre-computation points, $\mathcal{H} = \{h_1, \ldots, h_H\}$, where $0 < h_1 < \cdots < h_H = 1$, and evaluate each $\hat{l}_d$ only on the points in $\mathcal{H}$. For $1 \leq d \leq qD$ but $h \notin \mathcal{H}$ we linearly interpolate when $h_1 < h < h_H$,

$$
\hat{l}_d(h) \approx \hat{l}_d(h_i) + \frac{\hat{l}_d(h_{i+1}) - \hat{l}_d(h_i)}{h_{i+1} - h_i}(h - h_i)
$$

where $h_i < h < h_{i+1}$. For $h < h_1$ we compute the l-value directly from Eq 11.

In general both the computation time and the accuracy of the approximation tend to increase with the number of points H and the density of the pre-compute grid. In the mouse AIL data analysis, we use $H = 10^5$ and $h_1 = 10^{-20}$, and in the eQTLGen data analysis we use $H = 5 \times 10^4$ and $h_1 = 10^{-50}$. A naive means of assigning the values of $\mathcal{H}$ would be to create an evenly spaced grid between h_1 and 1, but this devotes few points, and little resolution, to small values of h. Instead we choose $\mathcal{H}$ to be the geometric sequence $h_i = h_1^{\frac{H-i}{H-1}}$ for $1 \leq i \leq H$. For the mouse AIL dataset, this results in 5,000 grid points for each order of

magnitude, scaled logarithmically, for our choice of h_1 and H, and it results in 1,000 grid points for each order of magnitude in the eQTLGen dataset.

Assessment of type 1 error with Monte Carlo p-values

We consider the null and alternative hypotheses given in Eqs 3 and 4 of the main text. Let T be any test statistic that is a function of $(\pi_1, \ldots, \pi_D)$ such that the hypothesis test can be performed by rejecting whenever $T < v$, where v is some suitably chosen threshold value. Suppose R i.i.d. Monte Carlo replicates are used to determine p-values (or thresholds) for T, where for $r = 1$ to R, Z^r is simulated as $Z^r \sim N_D(0, \Omega)$ and is then transformed to obtain the vector $\pi^r = (\pi_1^r, \ldots, \pi_D^r)$, where $\pi_i^r = 2\Phi(-|Z_i^r|)$, for $i = 1, \ldots D$. Then T is evaluated on each π^r to obtain R i.i.d. Monte Carlo replicates of T under the null hypothesis, call them $M_1, \ldots, M_R$. Let $M_{(1)}, \ldots, M_{(R)}$ denote the order statistics of $M_1, \ldots, M_R$. Then the Monte Carlo p-value for an observed value of the test statistic $T = t$ is given by $(k + 1)/(R + 1)$, where $k = 0$ if $t < M_{(1)}$, $k = R$ if $M_{(R)} \leq t$ and otherwise k is the unique value in $\{1, \ldots, R - 1\}$ such that $M_{(k)} \leq t < M_{(k+1)}$. For a given level α, if $(R + 1)\alpha$ happens to be an integer, then a test at level α can be obtained by rejecting when $k \leq (R + 1)\alpha - 1$. Under the null hypothesis, any ordering of the $R + 1$ values t and $M_1, \ldots, M_R$ is equally likely, so $P_0(\text{rejection}) = P_0(t < M_{((R+1)\alpha)}) = (R + 1)\alpha/(R + 1) = \alpha$. (If $(R + 1)\alpha$ does not happen to be an integer, then the slighly conservative test that rejects when $k \leq \text{floor}[(R + 1)\alpha - 1]$ can be used instead.) The type 1 error of such a procedure is automatically controlled when the R i.i.d. replicates are drawn under the null hypothesis.

In practice, the null distribution may have parameters that need to be estimated, e.g., Ω in the trans-eQTL setting we consider. In that case, correct type 1 error is not guaranteed and may need to be tested. In that case, suppose J simulation replicates are generated under the "true" distribution $Z \sim N(0, \Omega)$ and R Monte Carlo replicates are generated under the estimated distribution $Z \sim N(0, \hat{\Omega})$. Let the observed values of T among the simulation replicates be $S_1, \ldots, S_J$, and let the observed values of T among the Monte Carlo replicates be $M_1, \ldots, M_R$. For each $1 \leq j \leq J$, the Monte Carlo p-value assigned to S_j is $(k + 1)/(R + 1)$, where $k = 0$ if $S_j < M_{(1)}$, $k = R$ if $M_{(R)} \leq S_j$ and otherwise k is the unique value in $\{1, \ldots, R - 1\}$ such that $M_{(k)} \leq S_j < M_{(k+1)}$. Suppose we wish to use the results of the J simulation replicates and R Monte Carlo replicates to assess type 1 error at level α. We estimate type 1 error as $\tilde{N}/J$, where $\tilde{N}$ is defined to be the number of S_j's that are smaller than $M_{(c)}$, where $c = \text{floor}[(R + 1)\alpha]$. If we define $S_{(1)}, \ldots, S_{(J)}$ to be the order statistics of $S_1, \ldots, S_J$, and if we assume that the distribution of T is continuous, then we have that $P_0(\tilde{N} = k) = P_0(S_{(k)} < M_{(c)} \leq S_{(k+1)}) = P_0(A_{(c+k)} \text{is one of the M's}) \times P_0(\text{Among } A_{(1)}, \ldots, A_{(c+k)}, \text{ there are exactly } k \text{ S's} | A_{(c+k)} \text{is one of the M's})$, where we define $A_{(1)}, \ldots, A_{(R+J)}$

to be the order statistics of the combined sample consisting of $S_1, \ldots, S_J, M_1, \ldots, M_R$. Under the null hypothesis that $S_1, \ldots, S_J, M_1, \ldots, M_R$ is an i.i.d. sample, we have $P_0(A_{(c+k)}$is one of the M's$) = R/(R+J)$ (which is the chance that if you randomly draw an A, it is also an M), and P_0(Among $A_{(1)}, \ldots, A_{(c+k)}$, there are exactly k S's$|A_{(c+k)}$ is one of the M's$) = \binom{J}{k}\binom{R-1}{c-1}/\binom{J+R-1}{c+k-1}$. Then $P_0(\tilde{N} = k)$ is found to be $\binom{c+k-1}{k}\binom{J-k+R-c}{J-k}/\binom{J+R}{J}$.

From our formula for $P_0(\tilde{N} = k)$, we can calculate the expectation of the type 1 error estimate $\tilde{N}/J$ as $E(\tilde{N}/J) = c/(R+1)$. When $(R+1)\alpha$ is exactly an integer, this gives exactly α under the null hypothesis; otherwise it may differ from α by up to $1/(R+1)$ due to the discreteness. (For R of the size we consider this difference is negligible.) We obtain the variance of the type 1 error estimate $\tilde{N}/J$ as $\text{Var}(\tilde{N}/J) = c[(1-c/R)(R^2+R+RJ)+R+J+1]/[J(R+2)(R+1)^2]$. Assuming that both R and J are large, we obtain $\text{Var}(\tilde{N}/J) = \alpha(1-\alpha)(J^{-1}+R^{-1}) + O(1/R) + O(J/R^2) \approx \alpha(1-\alpha)(J^{-1}+R^{-1})$. An interesting implication of this formula comes from considering the following question: Suppose we had a fixed budget of $B = J + R$ replicates. How should we divide them up between Monte Carlo and simulation replicates to minimize the variance of the empirical type 1 error at level α? By a simple application of Lagrange multipliers, we obtain the minimum variance by setting $R = J = B/2$, i.e., by setting the number of Monte Carlo replicates equal to the number of simulation replicates. In that case, we obtain $\text{Var}(\tilde{N}/J) \approx 2\alpha(1-\alpha)/J$. This formula is used to determine the acceptance regions in main text Tables 1 and 2 for the statistics that use Monte Carlo p-values. To obtain the acceptance regions for the QQ-plots in Fig 1, S1 Fig and S2 Fig, we use the ELL method [4]. In Eq 3 of Weine et al., for F_0, we use the uniform distribution when ordinary p-values are plotted in the QQ-plot, and when Monte Carlo p-values are plotted in the QQ-plot, we instead use the distribution of $\tilde{N}/J$, which is obtained from the distribution of $\tilde{N}$ given in the previous paragraph, for F_0.

Generation of the correlation matrix for the simulations

We generated a random correlation matrix to serve as the $D \times D$ correlation matrix between traits. We created this matrix by generating a random orthogonal matrix to serve as eigenvectors and sampling a set of eigenvalues from an exponential distribution with rate parameter 0.05. A value of 1.0 was added to all the eigenvalues to prevent any values being close to zero. The eigenvectors and eigenvalues are combined to form a covariance matrix which we then convert to a correlation matrix.

Power Results

Table A. Power comparison for trans eQTL mapping when number of associated traits = 5

Method	Power (s.e.) at significance level			
	0.05	0.01	0.001	1e-04
Adelle q = 20%	0.717 (0.014)	0.501 (0.016)	0.281 (0.014)	0.113 (0.010)
Adelle q = 10%	0.719 (0.014)	0.511 (0.016)	0.289 (0.014)	*0.145 (0.011)
Adelle q = 5%	0.734 (0.014)	0.528 (0.016)	0.298 (0.014)	*0.148 (0.011)
Cauchy	*0.813 (0.012)	*0.643 (0.015)	*0.353 (0.015)	*0.172 (0.012)
Simes	*0.794 (0.013)	*0.618 (0.015)	*0.339 (0.015)	*0.167 (0.012)
min-P	*0.790 (0.013)	*0.612 (0.015)	*0.336 (0.015)	*0.166 (0.012)
G-null	0.608 (0.015)	0.344 (0.015)	0.131 (0.011)	0.035 (0.006)
Sum-χ^2	0.122 (0.010)	0.035 (0.006)	0.006 (0.002)	0.000 (0.000)
Archie	0.122 (0.010)	0.035 (0.006)	0.006 (0.002)	0.000 (0.000)
CPMA	0.062 (0.008)	0.020 (0.004)	0.002 (0.001)	0.000 (0.000)

Power is based on 1000 simulated replicates. s.e. denotes standard error of the estimate. A starred number denotes the highest power attained or power that is not significantly different (based on a z-test at level .05) from the highest power attained by any of the methods.

Table B. Power comparison for trans eQTL mapping when number of associated traits = 10

Method	Power (s.e.) at significance level			
	0.05	0.01	0.001	1e-04
Adelle q = 20%	*0.617 (0.015)	*0.369 (0.015)	*0.182 (0.012)	*0.063 (0.008)
Adelle q = 10%	*0.621 (0.015)	*0.378 (0.015)	*0.185 (0.012)	*0.080 (0.009)
Adelle q = 5%	*0.635 (0.015)	*0.391 (0.015)	*0.193 (0.012)	*0.085 (0.009)
Cauchy	*0.657 (0.015)	*0.405 (0.016)	*0.169 (0.012)	0.055 (0.007)
Simes	*0.632 (0.015)	*0.388 (0.015)	*0.162 (0.012)	0.055 (0.007)
min-P	*0.614 (0.015)	*0.381 (0.015)	*0.160 (0.012)	0.055 (0.007)
G-null	0.517 (0.016)	0.233 (0.013)	0.080 (0.009)	0.016 (0.004)
Sum-χ^2	0.148 (0.011)	0.042 (0.006)	0.008 (0.003)	0.000 (0.000)
Archie	0.148 (0.011)	0.043 (0.006)	0.008 (0.003)	0.000 (0.000)
CPMA	0.082 (0.009)	0.025 (0.005)	0.004 (0.002)	0.000 (0.000)

Power is based on 1000 simulated replicates. s.e. denotes standard error of the estimate. A starred number denotes the highest power attained or power that is not significantly different (based on a z-test at level .05) from the highest power attained by any of the methods.

Table C. Power comparison for trans eQTL mapping when number of associated traits = 20

Method	Power (s.e.) at significance level			
	0.05	0.01	0.001	1e-04
Adelle $q = 20\%$	*0.724 (0.014)	*0.488 (0.016)	*0.203 (0.013)	*0.063 (0.008)
Adelle $q = 10\%$	*0.733 (0.014)	*0.497 (0.016)	*0.207 (0.013)	*0.083 (0.009)
Adelle $q = 5\%$	*0.746 (0.014)	*0.520 (0.016)	*0.219 (0.013)	*0.083 (0.009)
Cauchy	0.675 (0.015)	0.371 (0.015)	0.127 (0.011)	0.040 (0.006)
Simes	0.617 (0.015)	0.343 (0.015)	0.122 (0.010)	0.040 (0.006)
min-P	0.597 (0.016)	0.336 (0.015)	0.120 (0.010)	0.039 (0.006)
G-null	0.641 (0.015)	0.296 (0.014)	0.082 (0.009)	0.014 (0.004)
Sum-χ^2	0.212 (0.013)	0.069 (0.008)	0.018 (0.004)	0.000 (0.000)
Archie	0.212 (0.013)	0.068 (0.008)	0.018 (0.004)	0.001 (0.000)
CPMA	0.119 (0.010)	0.036 (0.006)	0.007 (0.003)	0.000 (0.000)

Power is based on 1000 simulated replicates. s.e. denotes standard error of the estimate. A starred number denotes the highest power attained or power that is not significantly different (based on a z-test at level .05) from the highest power attained by any of the methods.

Table D. Power comparison for trans eQTL mapping when number of associated traits = 50

Method	Power (s.e.) at significance level			
	0.05	0.01	0.001	1e-04
Adelle $q = 20\%$	*0.737 (0.014)	*0.472 (0.016)	*0.204 (0.013)	0.047 (0.007)
Adelle $q = 10\%$	*0.741 (0.014)	*0.482 (0.016)	*0.216 (0.013)	*0.066 (0.008)
Adelle $q = 5\%$	*0.747 (0.014)	*0.499 (0.016)	*0.225 (0.013)	*0.071 (0.008)
Cauchy	0.519 (0.016)	0.230 (0.013)	0.040 (0.006)	0.009 (0.003)
Simes	0.461 (0.016)	0.196 (0.013)	0.038 (0.006)	0.009 (0.003)
min-P	0.433 (0.016)	0.185 (0.012)	0.038 (0.006)	0.009 (0.003)
G-null	0.664 (0.015)	0.347 (0.015)	0.087 (0.009)	0.013 (0.004)
Sum-χ^2	0.365 (0.015)	0.155 (0.011)	0.042 (0.006)	0.008 (0.003)
Archie	0.365 (0.015)	0.156 (0.011)	0.042 (0.006)	0.008 (0.003)
CPMA	0.221 (0.013)	0.084 (0.009)	0.025 (0.005)	0.004 (0.002)

Power is based on 1000 simulated replicates. s.e. denotes standard error of the estimate. A starred number denotes the highest power attained or power that is not significantly different (based on a z-test at level .05) from the highest power attained by any of the methods.

Table E. Power comparison for trans eQTL mapping when number of associated traits = 100

Method	Power (s.e.) at significance level			
	0.05	0.01	0.001	1e-04
Adelle $q = 20\%$	*0.914 (0.009)	*0.713 (0.014)	*0.394 (0.015)	0.137 (0.011)
Adelle $q = 10\%$	*0.913 (0.009)	*0.719 (0.014)	*0.403 (0.016)	*0.175 (0.012)
Adelle $q = 5\%$	*0.915 (0.009)	*0.731 (0.014)	*0.412 (0.016)	*0.180 (0.012)
Cauchy	0.570 (0.016)	0.223 (0.013)	0.051 (0.007)	0.011 (0.003)
Simes	0.482 (0.016)	0.196 (0.013)	0.049 (0.007)	0.011 (0.003)
min-P	0.443 (0.016)	0.191 (0.012)	0.048 (0.007)	0.011 (0.003)
G-null	0.870 (0.011)	0.609 (0.015)	0.262 (0.014)	0.063 (0.008)
Sum-χ^2	0.644 (0.015)	0.381 (0.015)	0.151 (0.011)	0.048 (0.007)
Archie	0.645 (0.015)	0.381 (0.015)	0.152 (0.011)	0.048 (0.007)
CPMA	0.469 (0.016)	0.236 (0.013)	0.081 (0.009)	0.029 (0.005)

Power is based on 1000 simulated replicates. s.e. denotes standard error of the estimate. A starred number denotes the highest power attained or power that is not significantly different (based on a z-test at level .05) from the highest power attained by any of the methods.

Table F. Power comparison for trans eQTL mapping when number of associated traits = 200

Method	Power (s.e.) at significance level			
	0.05	0.01	0.001	1e-04
Adelle $q = 20\%$	*0.691 (0.015)	*0.392 (0.015)	*0.142 (0.011)	*0.034 (0.006)
Adelle $q = 10\%$	*0.694 (0.015)	*0.392 (0.015)	*0.144 (0.011)	*0.043 (0.006)
Adelle $q = 5\%$	*0.691 (0.015)	*0.396 (0.015)	*0.138 (0.011)	*0.044 (0.006)
Cauchy	0.246 (0.014)	0.070 (0.008)	0.011 (0.003)	0.002 (0.001)
Simes	0.213 (0.013)	0.065 (0.008)	0.011 (0.003)	0.002 (0.001)
min-P	0.198 (0.013)	0.061 (0.008)	0.011 (0.003)	0.002 (0.001)
G-null	*0.680 (0.015)	*0.381 (0.015)	*0.136 (0.011)	0.024 (0.005)
Sum-χ^2	0.633 (0.015)	*0.369 (0.015)	*0.154 (0.011)	*0.044 (0.006)
Archie	0.633 (0.015)	*0.372 (0.015)	*0.154 (0.011)	*0.044 (0.006)
CPMA	0.487 (0.016)	0.266 (0.014)	0.088 (0.009)	*0.035 (0.006)

Power is based on 1000 simulated replicates. s.e. denotes standard error of the estimate. A starred number denotes the highest power attained or power that is not significantly different (based on a z-test at level .05) from the highest power attained by any of the methods.

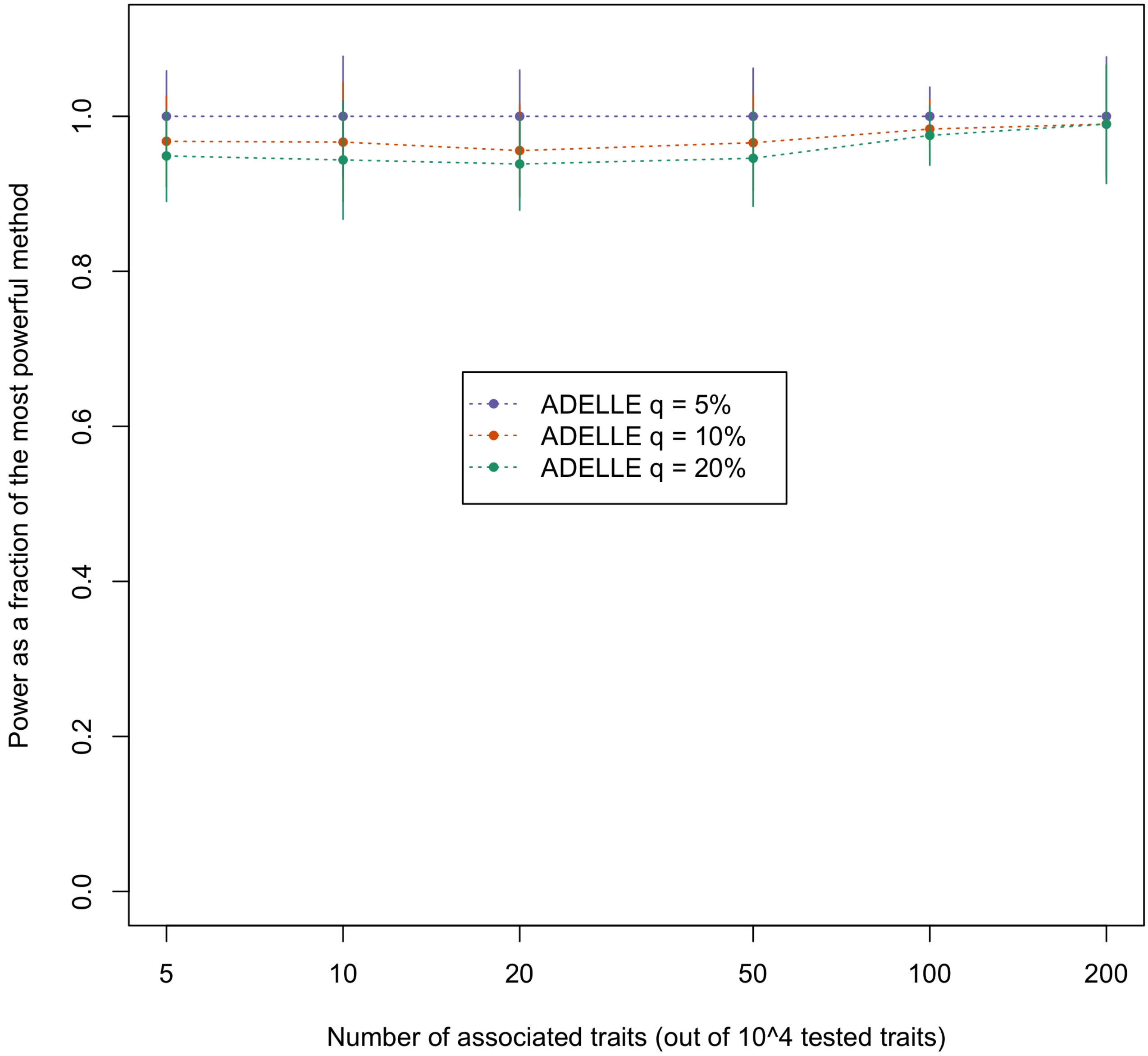

Fig. A. Relative power vs. number of associated traits for ADELLE with different choices of q
Relative power at significance level 0.01, based on 10^3 simulated replicates, is plotted against the number of associated traits out of 10^4 total traits tested, for ADELLE with $q = 5\%$, 10% or 20%, where q is the proportion of order statistics considered by ADELLE. For a given number of associated traits, relative power for a method is defined as its power divided by the maximum power achieved by any of the 3 depicted methods for that setting. For each point of the plot, the corresponding vertical bar represents the 95% confidence interval.

Table G Significant trans eQTL detections by ADELLE in a region of Chrom 12 in the mouse AIL dataset

rsID	Chrom	BP	Adelle pvalue	min-p	SNP annotation	cis eQTL	cis gene
rs262318378	12	72948661	1.0e-07	0.000372	downstream gene variant	No	NA
rs29176728	12	73012360	2.2e-06	0.006130	NA	No	NA
rs29223131	12	73320679	2.2e-06	0.001360	intron variant	No	NA
rs29197865	12	73409987	1.9e-06	0.002200	upstream gene variant	Yes	Ppm1a
rs213877154	12	73527315	2.2e-06	0.004000	NA	No	NA

min-p is the p-value Bonferroni corrected for the number of traits tested; SNP annotations are from [5]; cis eQTL is whether the SNP is also a cis eQTL; cis gene is the gene target of the cis eQTL.

References

1. Warton DI. Penalized normal likelihood and ridge regularization of correlation and covariance matrices. J Am Stat Assoc. 2008;103(481):340–349. doi:10.1198/016214508000000021.

2. Barnett I, Mukherjee R, Lin X. The generalized higher criticism for testing SNP-set effects in genetic association studies. J Am Stat Assoc. 2017;112(517):64–76. doi:10.1080/01621459.2016.1192039.

3. Sun R, Lin X. Genetic variant set-based tests using the generalized Berk–Jones statistic with application to a genome-wide association study of breast cancer. J Am Stat Assoc. 2020;115(531):1079–1091. doi:10.1080/01621459.2019.1660170.

4. Weine E, McPeek MS, Abney M. Application of equal local levels to improve Q-Q plot testing bands with R package qqconf. J Stat Softw. 2023;106(10):1–31. doi:10.18637/jss.v106.i10.

5. Gonzales NM, Seo J, Hernandez Cordero AI, St Pierre CL, Gregory JS, Distler MG, et al. Genome wide association analysis in a mouse advanced intercross line. Nat Commun. 2018;9:5162. doi:10.1038/s41467-018-07642-8.
