# Supplementary figures and images for: ADELLE: A global testing method for trans-eQTL mapping
Source: PLoS Genet. 2025 Jan 10;21(1):e1011563. doi: 10.1371/journal.pgen.1011563 (PMC11756770; doi:10.1371/journal.pgen.1011563)

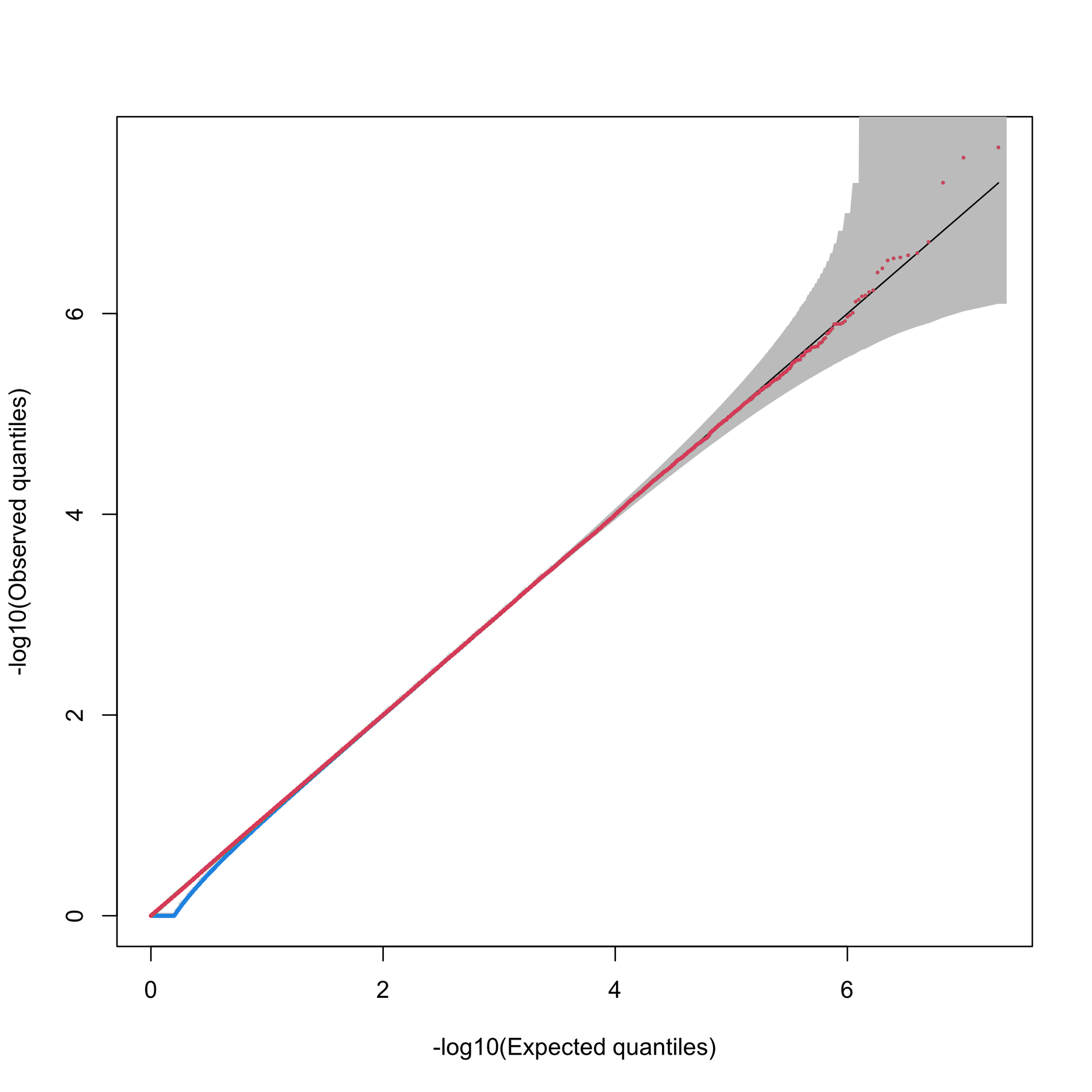

Supplement: S1 Fig — P-values from 20 million simulation replicates under the null hypothesis are shown for each method. The p-values from Min-P, Cauchy and Simes are in blue, red and green, respectively. Because the values are so similar, the 3 curves lie almost perfectly on top of one another, except for the large p-values where the Bonferroni correction used for Min-P is conservative. (TIF) [file pgen.1011563.s002.tif]

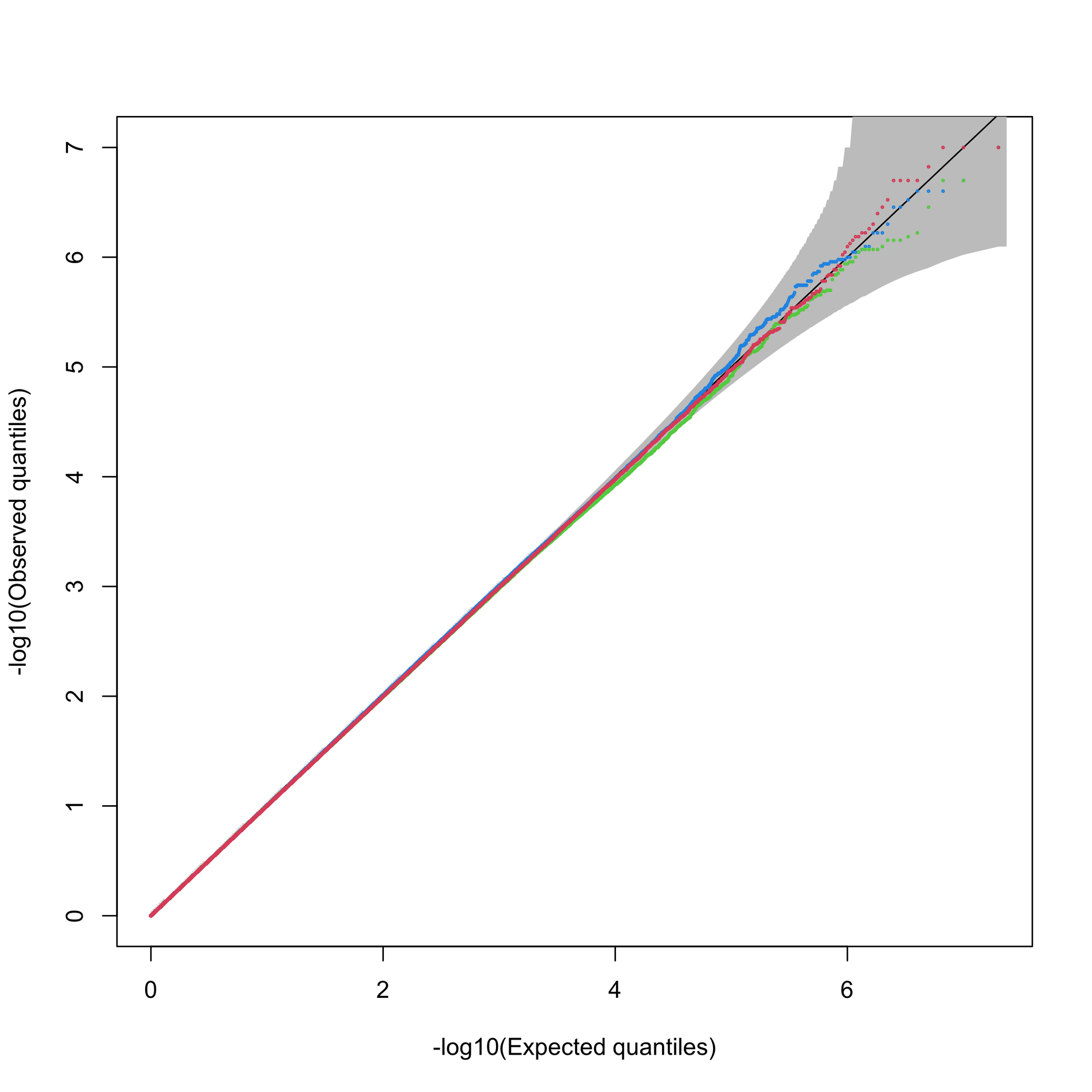

Supplement: S2 Fig — Empirical p-values based on 20 million simulation replicates and 20 million Monte Carlo replicates are shown for each method. The p-values from sum-χ2, G-Null and CPMA are in green, red, and blue, respectively. (TIF) [file pgen.1011563.s003.tif]
